# Calycosin inhibits the in vitro and in vivo growth of breast cancer cells through WDR7-7-GPR30 Signaling

**DOI:** 10.1186/s13046-017-0625-y

**Published:** 2017-11-02

**Authors:** Jing Tian, Yong Wang, Xing Zhang, Qianyao Ren, Rong Li, Yue Huang, Huiling Lu, Jian Chen

**Affiliations:** 1 Key Laboratory of Tumor Immunology and Microenvironmental Regulation, Guilin Medical University, Guilin, Guangxi 541004 China; 2 Department of Physiology, Guilin Medical University, Guilin, Guangxi China; 3 Department of Breast and Thyroid Surgery, First Affiliated Hospital of Guilin Medical University, Guilin, Guangxi China; 4 Department of Pathology and Physiopathology, Guilin Medical University, Guilin, Guangxi China

**Keywords:** Calycosin, Long non-coding RNA, WDR7-7, GPR30, Breast cancer

## Abstract

**Background:**

Clinically, breast cancer is generally classified into estrogen receptor-positive (ER+) or estrogen receptor-negative (ER−) subtypes. The phytoestrogen calycosin has been shown to inhibit the proliferation of ER+ cells, which may be mediated by a feedback loop that involves miR-375, RAS dexamethasone-induced 1 (RASD1), and ERα. However, how calycosin acts on ER− breast cancer cells remains unclear.

**Results:**

Here, we show that calycosin inhibited the proliferation of both ER− (MDA-MB-468 and SKBR3) and ER+ breast cancer cells (MCF-7 and T47D) and that these inhibitory effects were associated with the up-regulation of the long non-coding RNA (lncRNA) WDR7-7. For the first time, we demonstrate that the expression of WDR7-7 is reduced in breast cancer cell lines and that the overexpression of WDR7-7 inhibits growth through a mechanism that involves G-protein coupled estrogen receptor 30 (GPR30). Meanwhile, we show that calycosin stimulated the WDR7-7-GPR30 signaling pathway in MCF-7, T47D, MDA-MB-468, and SKBR3 breast cancer cells. In contrast, in MCF10A and GPR30-deficient MDA-MB-231 cells, due to a lack of WDR7-7-GPR30 for activation, calycosin failed to inhibit cell growth. Additionally, in all four GPR30-positive breast cancer lines, calycosin decreased the phosphorylation levels of SRC, EGFR, ERK1/2 and Akt, but the inhibition of WDR7-7 blocked these changes and increased proliferation. In mice bearing MCF-7 or SKBR3 xenografts, tumor growth was inhibited by calycosin, and changes in expression the levels of WDR7-7 and GPR30 in tumor tissues were similar to those in cultured MCF-7 and SKBR3 cells.

**Conclusions:**

These results suggest the possibility that calycosin inhibited the proliferation of breast cancer cells, at least partially, through WDR7-7-GPR30 signaling, which may explain why calycosin can exert inhibitory effects on ER− breast cancer.

**Electronic supplementary material:**

The online version of this article (10.1186/s13046-017-0625-y) contains supplementary material, which is available to authorized users.

## Background

Breast cancer is the second leading cause of cancer-related death among women in developed countries and ranks as the most frequent cause of cancer-related death in women in less developed regions of the world. Approximately 231,840 new cases of invasive breast cancer and 40,290 deaths were expected in the US women in 2015 [1]. Notably, the incidence rate of breast cancer in East Asian countries is lower than that in the US and in developed European countries, which may be due to higher amounts of phytoestrogen consumption in the former [2]. Phytoestrogens warrant investigation in breast cancer research because they are structurally similar to estrogen and exert estrogenic effects on breast cancer cells in low-estrogen environments but antiestrogenic effects in high-estrogen environments [3]. Plant-derived phytoestrogens can be divided into four main classes: isoflavones, coumestans, lignans, and stilbenes. Epidemiological studies and animal studies suggest that increased isoflavone intake from soy consumption may be associated with a decreased risk of developing breast cancer [4, 5].

However, in experimental studies using isoflavones for the prevention and treatment of human breast cancer, the data are conflicting [6, 7]. Clinically, breast cancer is generally classified into estrogen receptor-positive (ER+) or estrogen receptor-negative (ER−) subtypes. Isoflavones, such as genistein, daidzein, and formononetin, have been widely demonstrated to suppress the growth of ER+ breast cancer by binding to estrogen receptors [8–10]. The isoflavone calycosin (Molecular Formula: C_16_H_12_O_5_, Molecular Weight: 284.26, Fig. 5h) is a phytoestrogen and the main active component of *Radix Astragali*. Results from our previous study suggest that calycosin inhibits the proliferation of ER+ breast cancer cells and that miR-375, RAS dexamethasone-induced 1 (RASD1), and ER may be involved in this process [11, 12]. Unexpectedly, we found that calycosin also successfully inhibited the proliferation of ER− breast cancer cells in low-serum medium, although the effects were not as pronounced as ER+ breast cancer cells. These results indicate that in addition to ER-mediated signaling pathways, non-ER-mediated pathways are also involved in the calycosin-mediated regulation of breast cancer proliferation. Given the more aggressive biology of ER− breast cancers, calycosin treatment may result in better clinical outcomes than other phytoestrogens. Therefore, we focused on the antitumor effect of isoflavones against ER− breast cancer, the possible differences between ER+ and ER− breast cancer cells after treatment with calycosin, and the role of non-ER-mediated signaling in these effects.

The understanding of the importance of long ncRNAs (lncRNAs), a new class of non-coding RNAs (ncRNAs), in tumorigenesis is increasing. Recent evidence has suggested that lncRNA-mediated biology has a central role in cancer progression [13–15]. In our preliminary screen of lncRNAs in SKBR3 breast cancer cells, we saw that calycosin increased the expression of several lncRNAs, including WDR7-7, TTC21B-AS1, and CTA-384D8.34. Since WDR7-7 showed the greatest average increase in expression, we chose to examine the role of WDR7-7 in breast cancer carcinogenesis.

The target of WDR7-7, G-protein coupled estrogen receptor 30 (GPR30, formerly known as GPER), was present in both ER+ and ER− breast cancer cells, as confirmed by a luciferase reporter assay. Interestingly, GPR30 is an alternative ER that functions independently of the classical ER pathway and plays a key role in non-estrogen receptor-mediated pathways [16]. Several studies have shown that the inhibition of GPR30 signaling can elicit inhibitory effects on the growth of ER− breast cancer cells, but other studies show the opposite effect [17–20]. The effects of GPR30 on the proliferation of ER− breast cancer cells and the downstream signaling mechanisms of GPR30 urgently require further investigation. Therefore, in this study, we aimed to identify the function of GPR30 in breast cancer and the association between WDR7-7 and GPR30 signaling. In addition, we examined whether the WDR7-7-mediated regulation of the GPR30 pathway might be involved in the inhibitory effect induced by calycosin on breast cancer cells, especially in ER− breast cancer cells.

## Methods

### Experimental design

This study was composed of three parts: in vitro experiments in MCF-7, T47D, SKBR3, MDA-MB-468, MDA-MB-231, and MCF10A cells; in vitro analysis of breast cancer and paracancerous tissues samples; and in vivo experiments in nude mice bearing MCF-7-xenografts and SKBR3-xenografts.

For in vitro experiments, MCF-7, T47D, SKBR3, MDA-MB-468, MDA-MB-231, and MCF10A cells were treated with calycosin (Sigma, St. Louis, MO, USA, purity >98%) at concentrations of 1–32 μM. Then, cell growth was measured using a CCK-8 assay, BrdU assay, and colony formation assay. SKBR3 cells were treated with 16 μM calycosin for 48 h in a microarray screen to identify the lncRNAs that are specially regulated by calycosin. The target gene of WDR7-7, GPR30, was confirmed using luciferase reporter assays and quantitative real-time polymerase chain reaction (qRT-PCR). In the next set of experiments, MCF-7, T47D, SKBR3, MDA-MB-468, MDA-MB-231, and MCF10A cells were treated with calycosin (16 μM); then, the mRNA and proteins expression levels (WDR7-7, GPR30, p-SRC, phosphorylated epidermal growth factor receptor (p-EGFR), phosphorylated protein kinase B (p-Akt), and phosphorylated extracellular signal-regulated kinases (p-ERK1/2)) were measured using qRT-PCR and Western blotting. In some experiments, cells were pretreated for 20 h with the pCDNA3.1-WDR7-7 plasmid constructs, pCDNA3.1(+) vector, or WDR7-7 shRNA (100 nM; XuanC Bio, Nanning, Guangxi, China) prior to treatment with calycosin (16 μM).

For patients and specimen experiments, breast cancer and paracancerous tissues were used for immunohistochemical (IHC) staining and Western blotting analysis. Tissues were collected from breast cancer patients who underwent curative resection between 2013 and 2016 at the Department of Breast and Thyroid Surgery, First Affiliated Hospital of Guilin Medical University. None of the patients had received chemotherapy or radiotherapy before surgery. The study protocols were approved by the hospital ethics committee and the study was conducted according to the principles of the Declaration of Helsinki. All patients provided written informed consent.

For the in vivo experiments, all procedures were approved by the Animal Research Ethics Committee of Guilin Medical University. MCF-7 or SKBR3 cells were implanted subcutaneously into female nude mice (6–8 weeks old, *n* = 6 mice per condition). When the tumors reached a volume of 0.2 cm^3^ (approximately 10 days later), the mice were treated with calycosin (55 mg/kg/day), calycosin and pCDNA3.1-WDR7-7, or calycosin and WDR7-7 shRNA for 20 consecutive days.

### Cell culture and counting

MCF-7, T47D, SKBR3, MDA-MB-468, MDA-MB-231, and MCF10A cells were obtained from the Shanghai Institute of Cell Biology of the Chinese Academy of Sciences (Shanghai, China) and cultured in a humidified atmosphere with 5% CO_2_ at 37 °C. MCF-7, T47D, MDA-MB-468, MDA-MB-231, and SKBR3 cells were cultured in Roswell Park Memorial Institute (RPMI)-1640 media (Invitrogen, Carlsbad, CA, USA) supplemented with 10% FBS and 1% penicillin-streptomycin. MCF10A cells were cultured in DMEM-F12 (Invitrogen) containing 5% horse serum (HS, Invitrogen), 0.5 μg/ml hydrocortisone (Sigma), 10 μg/mL insulin (Sigma), 0.02 μg/ml EGF (Pepro Tech, Rocky Hill, NJ, USA), 0.1 μg/mL cholera toxin (Sigma), and penicillin-streptomycin. All cell lines were cultured in low-serum medium (1% FBS or HS) for 24 h prior to the experiments. Cell proliferation was examined using a CCK 8 kit (Sigma) and a BrdU kit (Roche Applied Science, Indianapolis, IN, USA) according to the manufacturer’s instructions. For colony formation assays, the cells were seeded in six-well plates. After incubation for 10, 14, and 18 days, the cell colonies were stained with 0.1% (*w*/*v*) crystal violet, photographed and then counted. Colony-forming efficiency (CFE) was calculated by the number of colonies formed divided by the total number of cells plated and is expressed as a percentage.

### Microarray of lncRNAs

Total RNA (including small RNAs) was extracted from SKBR3 cells using a miRNeasy Mini Kit (Qiagen, Valencia, CA, USA). RNA amplification and labeling were completed using the Amino Allyl MessageAmp II aRNA Amplification Kit (Ambion, Austin, TX, USA). Labeled cDNA was hybridized to a human lncRNA OneArray Plus Microarray (Phalanx Biotech Group, Hsinchu, Taiwan). After washing the slides, the arrays were scanned by an Agilent scanner (Agilent Technologies, Palo Alto, CA, USA), and data on the differential expression of lncRNAs were generated by Agilent Feature Extraction software (version 11.0.1.1).

### Gene function analysis

To identify the functions of lncRNAs, the predicted target genes of differentially expressed lncRNAs were added to the Database for Annotation, Integrated Discovery, and Visualization. A GO analysis was performed to analyze the functions represented in the lncRNA profile (XuanC Bio). Furthermore, the significant pathways of these differentially expressed lncRNAs were determined by the Kyoto Encyclopedia of Genes and Genomes database.

### Luciferase reporter assay

Lipofectamine 2000 (Invitrogen) was used to co-transfect MCF-7 and SKBR3 cells with a luciferase reporter plasmid (Promega, Madison, WI, USA) containing the wild-type or mutated 3′ UTR of the GPR30 gene downstream of the *Renilla* luciferase gene with a plasmid encoding hsa-WDR7-7 (XuanC Bio). After 48 h, luciferase activity was measured using a dual-luciferase assay system (Promega) and normalized to the activity of the *Renilla* luciferase internal control for transfection efficiency.

### Tumor samples and histological examination

Sections of paraffin-embedded breast cancer specimens were subjected to HE and IHC staining. For IHC, the sections were deparaffinized, hydrated, and immersed in 1% hydrogen peroxide in methanol for 30 min to block the endogenous peroxidase activity. The sections were incubated with rabbit anti-GPR30 polyclonal antibody (Abcam, Cambridge, Cambridgeshire, UK, diluted 1:250) overnight at 4 °C. After being washed with PBS, the sections were incubated with biotinylated secondary antibody (diluted 1:100) for 30 min at 37 °C, followed by exposure to horseradish peroxidase-conjugated goat anti-rabbit IgG for 20 min at 37 °C. The immunoreactive signal was visualized by the DAB detection system.

### Transfection

Lipofectamine 2000 (Invitrogen) was used to transfect MCF-7, T47D, SKBR3, MDA-MB-468, MDA-MB-231, and MCF10A cells with hsa-miR-375, pCDNA3.1-WDR7-7, miR-375 siRNA, or WDR7-7 shRNA (XuanC Bio).

### qRT-PCR

Total RNA was extracted using TRIzol reagent and reverse transcribed into cDNA using a Revert Aid First Strand cDNA Synthesis Kit (Fermentas, Hanover, MD, USA). The relative expression levels of *miR-375*, *WDR7-7*, and *GPR30* were measured by qRT-PCR using specific primers (Additional file 1: Table S1) and the SYBR Green qPCR Master Mix (Fermentas). The data were calculated using ABI 7500 software v2.0.1 (Applied Biosystems, Waltham, MA, USA). The expression levels of *GPR30* and *WDR7-7* were normalized to *β-actin* expression, and the expression level of *miR-375* was normalized to U6 snRNA.

### Western blotting

Proteins were extracted from tissues or cells using RIPA buffer (Beyotime, Nanjing, Jiangsu, China), separated by SDS-PAGE, and transferred onto polyvinylidene difluoride membranes (Millipore, Bedford, MA, USA). The membranes were incubated with primary antibodies (Sigma, diluted 1:500 to 1:1000) against the following proteins: ERα, RASD1, β-actin, GPR30, p-SRC, SRC, p-EGFR, EGFR, p-ERK1/2, ERK1/2, p-Akt, and Akt. The blots were washed three times, incubated with the appropriate secondary antibodies (Beyotime), and then visualized with enhanced chemiluminescence reagents (Beyotime). Band intensities were quantified using Image-Pro Plus 5.02 software (Media Cybernetics, Bethesda, MD, USA). The intensities of the ERα, RASD1, and GPR30 bands were normalized to the intensity of the corresponding β-actin band, and the intensity of phosphorylated proteins was normalized to that of the corresponding unphosphorylated proteins.

### Tumor xenografts

Mice were injected subcutaneously with 1 × 10^7^ MCF-7 or SKBR3 cells. When the tumor reached 2 cm in diameter, it was divided into pieces approximately 1 mm × 1 mm × 1 mm. These pieces were implanted into 24 recipient mice. When the tumors reached a size of 0.2 cm^3^, the mice were treated with calycosin (0, 55 mg/kg), 55 mg/kg calycosin and pCDNA3.1-WDR7-7, or 55 mg/kg calycosin and WDR7-7 shRNA for 20 days. Tumor growth was examined every 4 days and the tumors were harvested after 30 days to determine the expression levels of WDR7-7 and GPR30 using qRT-PCR and Western blotting.

### Statistical analysis

The results are expressed as the means ± standard deviations. Comparisons between multiple groups were made using a one-way analysis of variance (ANOVA), followed by Tukey’s post hoc test. Statistical analyses were conducted with SPSS 19.0 software (IBM, Chicago, IL, USA). Significance was defined as *p* < 0.05.

## Results

### Concentration- and cell type-dependent effects of calycosin on cell proliferation

**Fig. 1:**
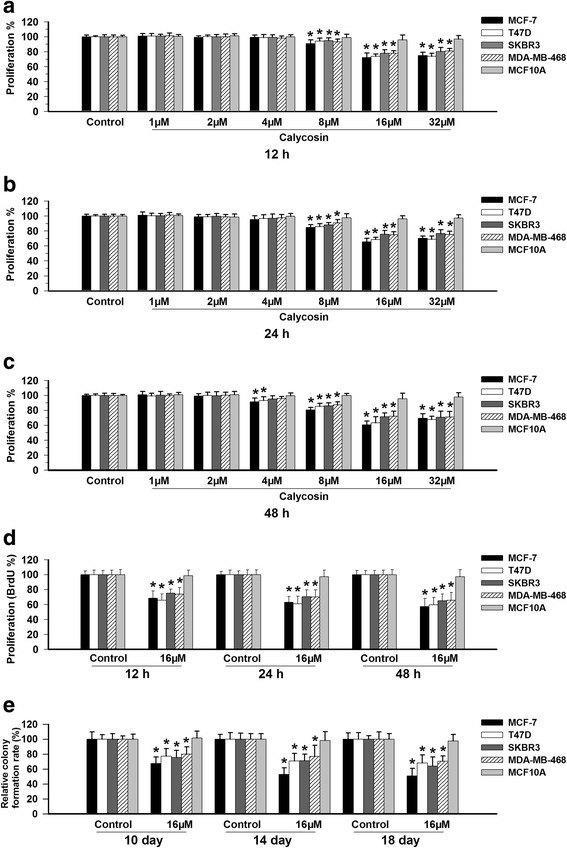
The effects of calycosin on the proliferation of breast cancer cells and MCF10A cells. MCF-7, T47D, SKBR3, MDA-MB-468 and MCF10A cells were treated for 12, 24, or 48 h with calycosin (1–32 μM); then, cell proliferation was quantified using (**a**-**c**) the CCK-8 assay, (**d**) BrdU assay, and (**e**) colony formation assay. The results are from three independent experiments that were each conducted in triplicate. *p < 0.05 vs. control (0 μM)

The anti-proliferative effects of calycosin were assessed by incubating MCF-7, T47D, SKBR3, MDA-MB-468, MDA-MB-231, and MCF10A cells with different concentrations of calycosin for 12, 24, and 48 h, followed by analysis with the CCK-8 and BrdU assays. Treatment with 4–16 μM calycosin inhibited cell proliferation in a concentration-dependent manner in the MCF-7, T47D, SKBR3, and MDA-MB-468 breast cancer cell lines (*p* < 0.05; Fig. 1a-d). This inhibitory effect was much greater in ER+ breast cancer cells (MCF-7 and T47D) than in ER− breast cancer cells (MDA-MB-468 and SKBR3). Notably, calycosin did not affect the proliferation of the ER− normal human breast epithelial cell line MCF10A or the GPR30-deficient ER− MDA-MB-231 cells (Additional file 5: Figure S3A-B), even at the highest concentration. To confirm the anti-proliferative effects of calycosin, we assessed the CFE of the five breast cancer cell lines and the normal MCF10A cell line (Fig. 1e, Additional file 5: Figure S3C). Consistent with the CCK-8 and BrdU assays results, a reduced CFE comparable to that of the control was observed in all four GPR30-positive breast cancer cells but not in MDA-MB-231 cells or in the normal breast epithelial MCF10A cell line.

### Involvement of the miR-375-ERα feedback loop in calycosin-regulated proliferation in MCF-7 and T47D cells

**Fig. 2:**
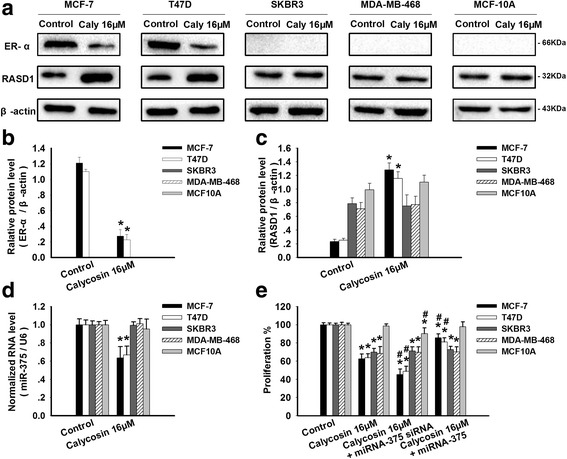
Calycosin inhibits cell proliferation and survival by regulating ERα, RASD1, and miR-375 in ER+ breast cancer cells. MCF-7, T47D, SKBR3, MDA-MB-468, and MCF10A cells were treated for 48 h with calycosin (0, 16 μM), 16 μM calycosin and premiR-375, or 16 μM calycosin and miR-375 siRNA. The protein and mRNA expression levels of (**a**, **b**) ERα, (**a**, **c**) RASD1, and (**d**) miR-375 were determined using Western blotting and qRT-PCR. The ERα and RASD1 protein expression levels were normalized to β-actin expression, the internal control. Transcript levels were normalized to those of U6 snRNA in the case of miR-375. **e** Cell proliferation was analyzed using the CCK-8 assay. Representative data from three independent experiments are shown. *p < 0.05 vs. control (0 μM); #*p* < 0.05 vs. 16 μM calycosin alone

Several studies and our previous data identified the existence of a miR-375-ERα feedback loop in ER+ breast cancer cells [11, 21]. To examine whether the anti-proliferative effects of calycosin were related to the feedback loop, MCF-7, T47D, MDA-MB-468, SKBR3, and MCF10A cells were pretreated with pre-miR-375 or miR-375 siRNA prior to treatment with calycosin (16 μM). In MCF-7 and T47D cells, calycosin down-regulated miR-375 and ERα expression and up-regulated RASD1 expression according to the qRT-PCR and Western blotting results (*p* < 0.05; Fig. 2a-d). The same treatment did not affect miR-375 or RASD1 expression in the ER− MDA-MB-468 or SKBR3 breast cancer cells or in the MCF10A cells (Fig. 2a-d). Pretreatment with miR-375 siRNA further increased the anti-proliferative effects of calycosin in MCF-7 and T47D cells, but not in MDA-MB-468, SKBR3, or MCF10A cells (*p* < 0.05 vs. 16 μM calycosin alone; Fig. 2e). In contrast, the overexpression of miR-375 attenuated the anti-proliferative effects of calycosin in MCF-7 and T47D cells (*p* < 0.05 vs. 16 μM calycosin alone; Fig. 2e). These data confirm the existence of the miR-375-ERα feedback loop via RASD1 in MCF-7 and T47D cells and its role in the calycosin-induced inhibitory effects on ER+ breast cancer cells rather than ER− cells.

### The expression of WDR7-7 and GPR30 in breast cancer cells and tissues

**Fig. 3:**
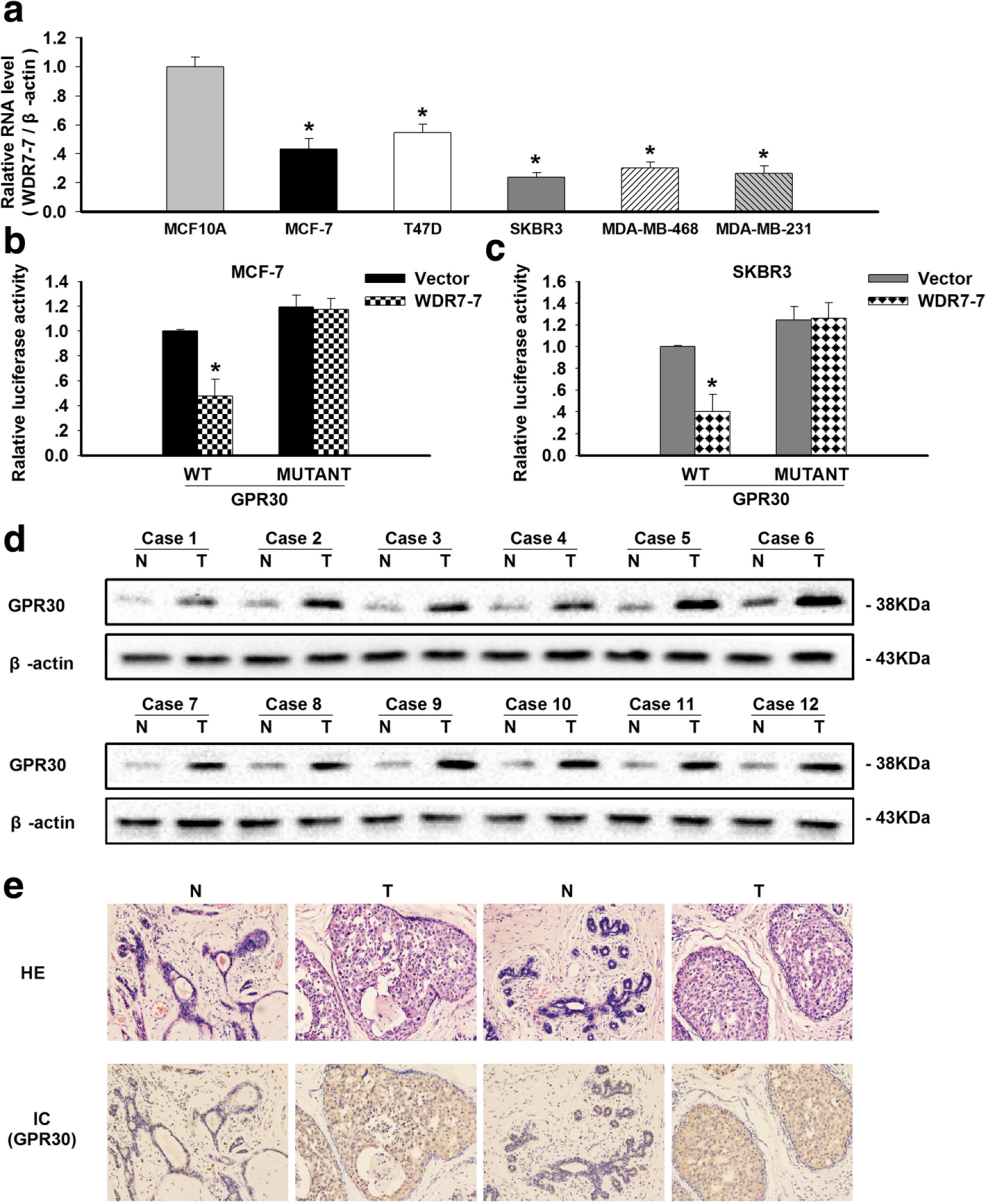
The expression levels of WDR7-7 and GPR30 in breast cancer cells and the association between WDR7-7 and GPR30. **a** The down-regulated expression of WDR7-7 was further confirmed in five breast cancer cell lines (MCF-7, T47D, SKBR3, MDA-MB-468, and MDA-MB-231). Representative data from three independent experiments are shown. *p < 0.05 vs. MCF10A cells. **b**-**c** The luciferase activity of MCF-7 and SKBR3 cells co-transfected with either a plasmid encoding wild-type or mutated 3′ UTR of GPR30 and either an empty vector or a plasmid encoding WDR7-7. Renilla luciferase activity was normalized to firefly luciferase activity and was plotted as relative luciferase activity. *p < 0.05 vs. vector. GPR30 protein expression levels in human breast cancer tissues (T) and adjacent normal tissues (N) were examined by (**d**) Western blot analyses with β-actin as the internal control; (**e**) HE staining and GPR30 immunohistochemistry (200× magnification)

In addition to the miR-375-ERα feedback loop, we explored other possible mechanisms by which calycosin inhibits the proliferation of breast cancer cells, especially in ER− subtypes. The expression profiles of lncRNAs in the SKBR3 human breast cancer cells were measured via microarray following treatment with calycosin. Calycosin significantly up-regulated 152 lncRNAs in SKBR3 cells (average fold change >2; *p* < 0.05), with the greatest effect on the lncRNA WDR7-7 (Additional file 2: Table S2). Next, the reduced expression of WDR7-7 was confirmed in five breast cancer cell lines (ER+ and ER−) (*p* < 0.05; Fig. 3a). Based on the microarray profiling and gene function analysis, we focused on GPR30 as a potential WDR7-7 target. Furthermore, luciferase reporter assays confirmed GPR30 as a functional target of WDR7-7 in both MCF-7 and SKBR3 cells (Fig. 3b-c). In breast cancer tissues, GPR30 expression mainly localized to the cytoplasm and the cell membrane (Fig. 3e). The protein expression levels of GPR30 in breast cancer tissues increased compared with those of normal breast tissues, as determined by Western blotting (*p* < 0.05; Fig. 3d).

### Involvement of the WDR7-7-GPR30 signaling pathway in the proliferation of breast cancer cells

**Fig. 4:**
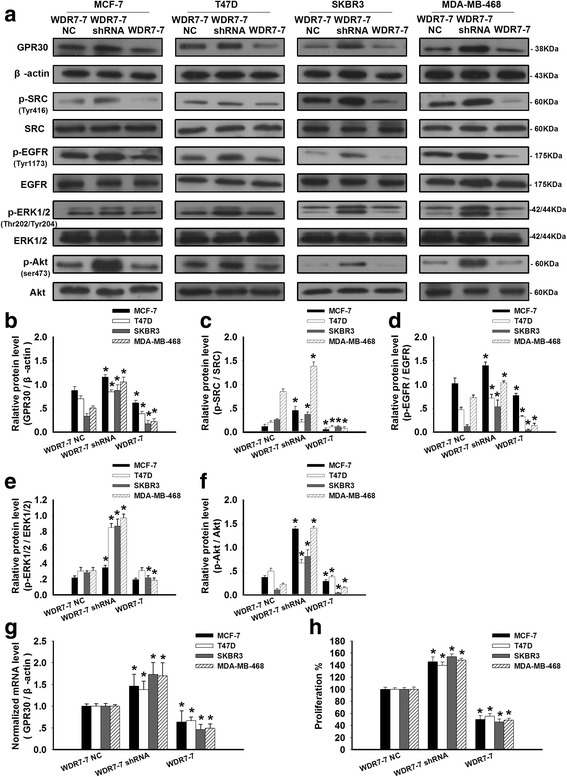
Regulation of GPR30 signaling by WDR7-7 to inhibit proliferation in breast cancer cells. The effects of WDR7-7 on GPR30 signaling were detected by (**a**) Western blotting in MCF-7, T47D, SKBR3, and MDA-MB-468 cells. The knock-down or overexpression of WDR7-7 regulated (**b**) GPR30 protein expression levels and the phosphorylation of (**c**) SRC, (**d**) EGFR, (**e**) ERK1/2, and (**f**) Akt. The expression level of GPR30 was normalized to β-actin as the internal control, and the phosphorylation levels SRC, EGFR, ERK1/2, and Akt used the corresponding total protein as the internal control in the same sample. (**g**) The expression levels of GPR30 mRNA after the overexpression or knock-down of WDR7-7 were determined using qRT-PCR. **h** The knock-down or overexpression of WDR7-7 regulated cell proliferation, as evidenced by the CCK-8 assay results. Representative data from three independent experiments are shown. *p < 0.05 vs. WDR7-7 control

After demonstrating the relationship between WDR7-7 and GPR30 expression in breast cancer cells, we investigated the association between the WDR7-7-GPR30 signaling pathway and proliferation in breast cancer cells and the mechanism of this effect. Pretreatment with pCDNA3.1-WDR7-7 or WDR7-7 shRNA increased or reduced WDR7-7 levels, respectively, in breast cancer cells (Additional file 3: Figure S1). We found that silencing WDR7-7 with specific shRNAs increased GPR30 levels in both ER+ (MCF-7, T47D) and ER− breast cancer cells (SKBR3, MDA-MB-468), contributing to the promotion of cell growth (*p* < 0.05; Fig. 4a-b, g-h). In contrast, the overexpression of WDR7-7 reduced GPR30 expression and decreased cell survival (*p* < 0.05; Fig. 4a-b, g-h). These data confirm the existence of a WDR7-7-GPR30 signaling pathway in breast cancer cells and suggest that this pathway plays a role in cancer growth.

Previous studies have linked GPR30 expression to the non-receptor tyrosine kinase (Src)-dependent activation of EGFR signaling in a variety of estrogen-responsive cancer cells [22]. Activation of the Src/EGFR pathway plays a role in the promotion of cell transformation, proliferation and survival [23]. Consistent with the increased cell viability in breast cancer cells, pretreatment with WDR7-7 shRNA significantly triggered the up-regulation of GPR30 and the phosphorylation of SRC and EGFR and subsequently increased the phosphorylation levels of ERK1/2 and Akt in all four breast cancer cells (*p* < 0.05; Fig. 4a-f). In contrast, WDR7-7 overexpression led to the inhibition of the GPR30-mediated phosphorylation of SRC, EGFR, ERK1/2, and Akt, ultimately resulting in reduced cell growth. Together, these findings suggest that SRC, EGFR, ERK1/2, and Akt, which are key regulators of cell proliferation and survival in breast cancer cells, act as downstream effectors of the WDR7-7-GPR30 pathway, especially in ER− cells due to the absence of an ER-mediated pathway.

### The effects of calycosin on the WDR7-7-GPR30 signaling pathway

**Fig. 5:**
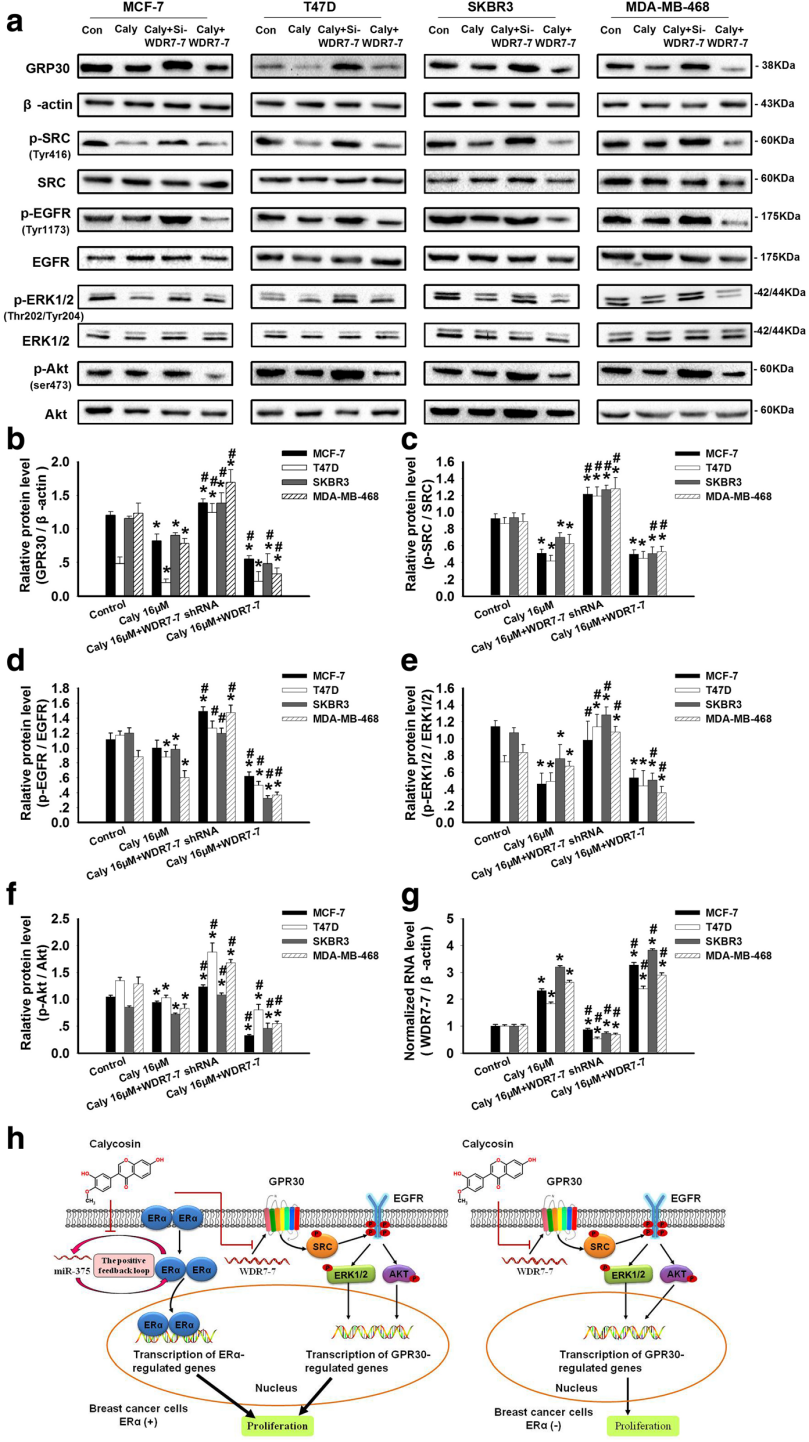
Calycosin inhibits cell proliferation by regulating WDR7-7-GPR30 signaling. MCF-7, T47D, SKBR3, and MDA-MB-468 cells were treated for 48 h with 16 μM calycosin, 16 μM calycosin and pCDNA3.1-WDR7-7, or 16 μM calycosin and WDR7-7 shRNA. **a**-**f** Western blotting was used to determine the protein expression levels of GPR30, p-SRC, p-EGFR, p-ERK1/2, and p-Akt. The protein expression levels were determined using Western blotting and normalized to either β-actin or the corresponding total protein as the internal control. **g** The expression levels of WDR7-7 were normalized to those of β-actin. Representative data from three independent experiments are shown. *p < 0.05 vs. control (0 μM); #*p* < 0.05 vs. 16 μM calycosin alone. **h** Schematic illustration of the proposed mechanism by which calycosin inhibits proliferation in breast cancer cells. This activity depends on WDR7-7-GPR30 signaling and a feedback loop that involves miR-375, RASD1, and ERα

Calycosin up-regulated WDR7-7 expression in MCF-7, T47D, SKBR3, MDA-MB-468, and MDA-MB-231 cells but not in MCF10A cells (*p* < 0.05; Fig. 5g, Additional file 4: Figure S2A, Additional file 5: Figure S3D). Subsequently, decreased GPR30 expression was found in MCF-7, T47D, SKBR3, and MDA-MB-468 cells, but no change was observed in MCF10A cells (*p* < 0.05; Fig. 5a-b, Additional file 4: Figure S2B). In addition to the inactivation of GPR30, calycosin down-regulated the phosphorylation levels of SRC, EGFR, ERK1/2, and Akt in both ER+ (MCF-7, T47D) and ER− cells (SKBR3, MDA-MB-468) based on Western blotting assays (Fig. 5a-f). Meanwhile, pretreating cells with WDR7-7 shRNA reversed the calycosin-induced down-regulation of GPR30 in all four cell lines, followed by the increased phosphorylation of SRC, EGFR, ERK1/2, and Akt (*p* < 0.05 vs. 16 μM calycosin alone). In contrast, the overexpression of WDR7-7 led to a greater calycosin-induced down-regulation of GPR30, p-SRC, p-EGFR, p-ERK1/2, and p-Akt expression (*p* < 0.05 vs. 16 μM calycosin alone). However, calycosin did not affect the expression of p-SRC, p-EGFR, p-ERK1/2, or p-Akt in GPR30-deficient MDA-MB-231 cells (Additional file 5: Figure S3E-I). These results indicate that calycosin inhibited the WDR7-7-GPR30 signaling pathway in GPR30-positive breast cancer, which may be especially significant for ER− breast cancer cells.

### The effects of calycosin on tumor xenografts

**Fig. 6:**
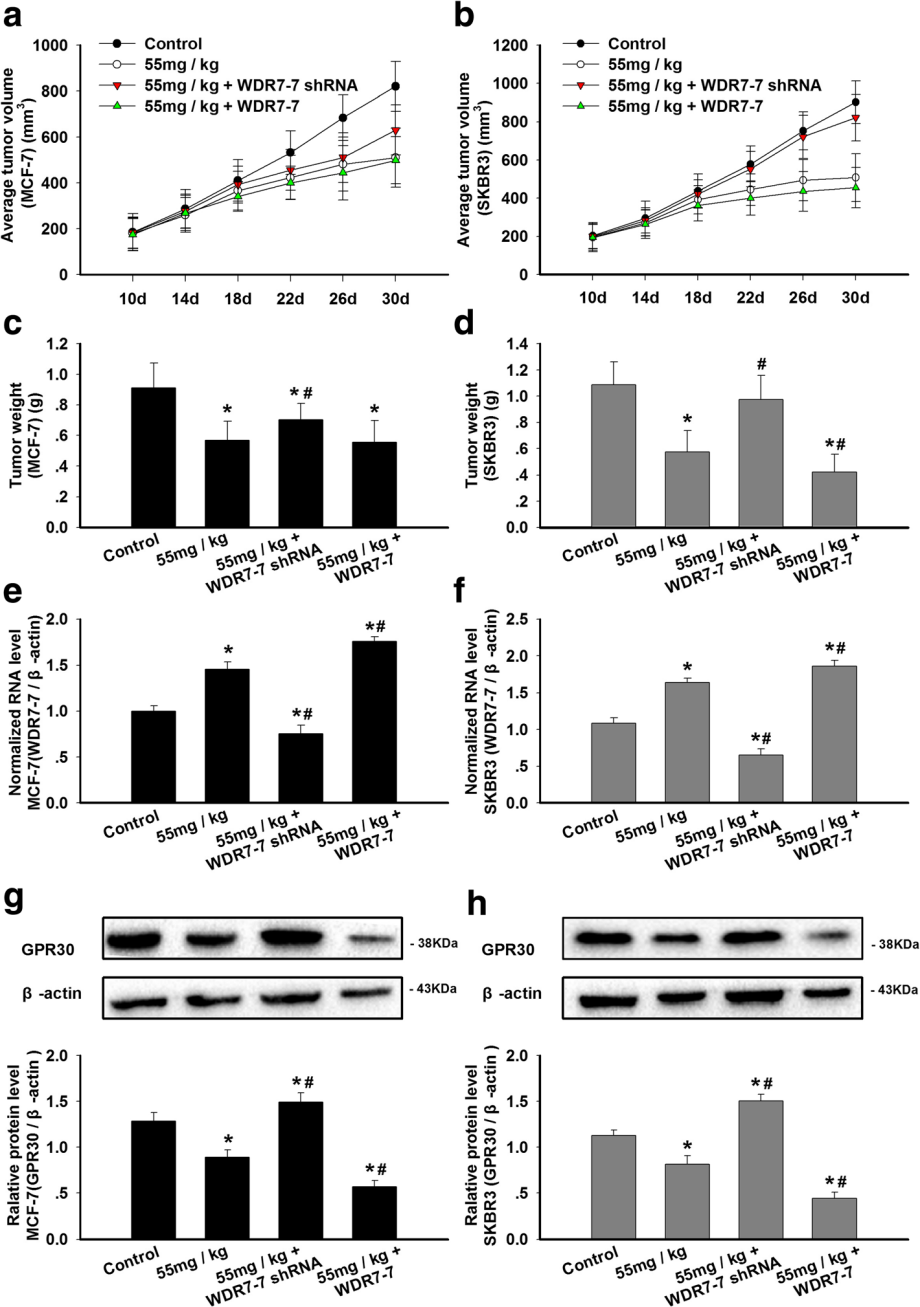
The effects of calycosin on xenograft tumor growth and the expression levels of WDR7-7 and GPR30 in tumor tissue. MCF-7 or SKBR3-xenografted mice were treated for 20 days with calycosin (0, 55 mg/kg), 55 mg/kg calycosin and pCDNA3.1-WDR7-7, or 55 mg/kg calycosin and WDR7-7 shRNA. **a**, **b** The tumor volume and (**c**, **d**) weight of MCF-7 or SKBR3 xenografts were measured (*n* = 6). *p < 0.05 vs. control; #*p* < 0.05 vs. 55 mg/kg calycosin. The expression levels of (**e**-**f**) WDR7-7 and **g**-**h** GPR30 in tumor tissues were determined using qRT-PCR or Western blotting. The expression levels of WDR7-7 and GPR30 were normalized to those of β-actin. Representative data from three independent experiments are shown. *p < 0.05 vs. control; #*p* < 0.05 vs. 55 mg/kg calycosin

Our observation that calycosin inhibited the proliferation of breast cancer cells in vitro led us to examine the anti-proliferative effects of calycosin in breast carcinoma tumor xenografts in vivo. Both tumor volume and weight in calycosin-treated MCF-7 and SKBR3 xenograft groups were significantly smaller than those of the control group (*p* < 0.05; Fig. 6a-d). Pretreatment with WDR7-7 shRNA antagonized this calycosin-induced decrease in tumor volume and weight, and the up-regulation of WDR7-7 expression further inhibited tumor growth. Likewise, it was found that calycosin significantly up-regulated WDR7-7 expression and down-regulated GPR30 expression in xenografts (*p* < 0.05; Fig. 6e-h). These effects were enhanced or abrogated by pretreating the animals with pCDNA3.1-WDR7-7 or WDR7-7 shRNA.

## Discussion

ERs are involved in the development and progression of estrogen-related cancers, such as breast cancer, ovarian cancer, and prostate cancer [24–26]. Evidently, some phytoestrogens are structurally similar to estrogen and may elicit inhibitory effects on tumor cell proliferation by binding ERs [27, 28]. Likewise, in this study, we demonstrated that calycosin successfully inhibited the growth of ER+ MCF-7 and T47D breast cancer cells, which was mediated by the inactivation of the miR-375-ERα feedback loop. Interestingly, calycosin also had an inhibitory effect on the viability of ER− MDA-MB-468 and SKBR3 cells but not on the proliferation of ER− MCF10A cells. This indicates that, other than normal human breast epithelial cells, calycosin mediates its effects on breast cancer at least in part through mechanisms that are independent of classical estrogen receptors.

In addition to ERs, studies have shown that GPR30, a G protein-coupled receptor, can also be activated by estrogen and play an important role in cancer development [29]. However, some controversy remains on whether GPR30 inhibits or promotes breast cancer development [30–32]. Here, we validated by IHC and Western blotting that GPR30 expression is up-regulated in breast cancer tissues, implying that activation of the GPR30 signaling pathway may be involved in the proliferation of breast cancer cells. However, less is known about the molecular basis of GPR30 in breast cancer and the possible interactions between phytoestrogens and the GPR30-mediated signaling pathway.

Microarray screening identified 152 lncRNAs in SKBR3 cells that were up-regulated by calycosin. The effect was greatest in the case of WDR7-7. Moreover, the down-regulation of WDR7-7 expression was confirmed in both breast cancer cells lines, including MCF-7, T47D, SKBR3, MDA-MB-468, and MDA-MB-231 cells. We found that the overexpression of WDR7-7 in breast cancer cells decreased proliferation, and that silencing WDR7-7 led to increased cell survival. In this regard, we believe that WDR7-7 may function as a tumor suppressor to inhibit cell growth in breast cancer. Interestingly, our luciferase reporter assays confirmed GPR30 as a target of WDR7-7 in breast cancer cells. We also found that the overexpression of WDR7-7 down-regulated GPR30 and that the shRNA-mediated knock down of WDR7-7 up-regulated GPR30. Therefore, we provide evidence that, in addition to the miR-375-ERα feedback loop in ER+ breast cancer cells, a negative relationship between WDR7-7 and GPR30 exists in both ER+ and ER− breast cancer cells.

In the present study, calycosin was found to down-regulate GPR30 by increasing WDR7-7 expression in all breast cancer cells, and the inhibition of WDR7-7 expression significantly reduced the antiproliferative effects of calycosin. MDA-MB-231 breast cancer cells lack GPR30 expression, so treatment with 16 μM calycosin did not suppress cell growth. Furthermore, the involvement of WDR7-7-GPR30 signaling was observed in vivo. Calycosin decreased tumor weight and volume in both ER+ MCF-7 and ER− SKBR3 xenografts, and in cancer tissues, calycosin increased the expression of WDR7-7 but decreased the expression of GPR30. Similarly, these effects were attenuated after pretreatment with a WDR7-7 inhibitor, confirming that the calycosin-induced inhibition of breast cancer cell proliferation involves the WDR7-7-GPR30 pathway. Additionally, the finding that calycosin inhibited both the miR-375-ERα feedback loop and the WDR7-7-GPR30 pathway in ER+ breast cancer cells may explain why ER+ breast cancer cells seem to be more sensitive to calycosin than ER− breast cancer cells. In ER− MCF10A cells, calycosin failed to trigger both pathways and, therefore, exerted no effect on cell growth.

Some studies have determined that GPR30 can be stimulated by estrogen to activate numerous cell signaling pathways through the transactivation of the EGFR/ERK pathway [33–35]. For example, in ER− breast cancer cells, GPR30 activates the tyrosine kinase SRC through the Gβγ subunit, which stimulates the autophosphorylation of EGFR to initiate MAP-kinase ERK1/2 and phosphatidylinositol 3 kinase (PI3K) signaling, ultimately inducing proliferation [36, 37]. As expected, we found that after reducing GPR30 expression, calycosin caused a decrease the phosphorylation levels of SRC, EGFR, ERK1/2, and Akt in breast cancer cell lines with and without ER expression (MCF-7, T47D, MDA-MB-468, and SKBR3), but calycosin did not affect the expression of p-SRC, p-EGFR, p-ERK1/2, or p-Akt in GPR30-deficient MDA-MB-231 cells. Pretreatment with a WDR7-7 inhibitor attenuated these effects in all four cell lines, but pretreatment with the pCDNA3.1-WDR7-7 vector promoted the calycosin-induced inhibition of these protein activities. These results suggest that SRC, EGFR, ERK1/2, and Akt help mediate the proliferation of breast cancer cells as downstream mediators of the WDR7-7-GPR30 pathway.

## Conclusions

Altogether, the present study provides in vitro and in vivo evidence for a better understanding of the novel mechanisms by which calycosin inhibits the proliferation of breast cancer cells, especially ER− breast cancer cells. These actions likely involve the WDR7-7-GPR30 pathway, which then inactivates the MAPK and PI3K/Akt pathways via down-regulation of SRC and EGFR (Fig. 5h). Therefore, WDR7-7 and GPR30 may be potential mediators of the anti-cancer activity of calycosin that function alongside traditional estrogen receptors and may serve as additional biological targets in estrogen-sensitive tumors.

## Additional files

Additional file 1: Table S1. The primer sequences. (DOCX 11 kb)

Additional file 2: Table S2. The effects of calycosin treatment on lncRNA profiles in SKBR3 cells. (DOC 37 kb)

Additional file 3: Figure S1. Relative WDR7-7 expression in breast cancer cells transfected with WDR7-7 control, WDR7-7 shRNA, or pCDNA3.1-WDR7-7 vector. Representative data from three independent experiments are shown. *p < 0.05 vs. WDR7-7 control. (JPEG 297 kb)

Additional file 4: Figure S2. The effects of calycosin on WDR7-7 and GPR30 expression in MCF10A cells. MCF10A cells were treated for 48 h with calycosin (0, 16 μM). (A) The transcript expression levels of WDR7-7 were determined using qRT-PCR with β-actin as the internal control. (B) The protein expression levels of GPR30 were determined using Western blotting and were normalized to those of β-actin. Representative data from three independent experiments are shown. (JPEG 267 kb)

Additional file 5: Figure S3. The effect of calycosin on the proliferation of MDA-MB-231 breast cancer cells and WDR7-7-GPR30 signaling. MDA-MB-231 cells were treated for 12, 24, or 48 h with calycosin (1–32 μM); then, cell proliferation was quantified using (A) the CCK-8 assay, (B) BrdU assay, and (C) colony formation assay. (D) The transcript expression levels of WDR7-7 were determined using qRT-PCR with β-actin as the internal control. (E-I) The phosphorylation levels of SRC, EGFR, ERK1/2, and Akt were determined using Western blotting with the corresponding total protein as the internal control. The results are from three independent experiments that were each conducted in triplicate. *p < 0.05 vs. control (0 μM). (JPEG 892 kb)
