# Correction to: Calycosin inhibits the in vitro and in vivo growth of breast cancer cells through WDR7-7-GPR30 Signaling

**DOI:** 10.1186/s13046-017-0660-8

**Published:** 2017-12-15

**Authors:** Jing Tian, Yong Wang, Xing Zhang, Qianyao Ren, Rong Li, Yue Huang, Huiling Lu, Jian Chen

**Affiliations:** 1 Key Laboratory of Tumor Immunology and Microenvironmental Regulation, Guilin Medical University, Guilin, Guangxi 541004 China; 2 Department of Physiology, Guilin Medical University, Guilin, Guangxi China; 3 Department of Breast and Thyroid Surgery, First Affiliated Hospital of Guilin Medical University, Guilin, Guangxi China; 4 Department of Pathology and Physiopathology, Guilin Medical University, Guilin, Guangxi China

## Correction

In the publication of this article [1], the molecule weight of GPR30 in figures was incorrectly, this should have been 55 kDa, and not 38 kDa.

This has now been included in this erratum.
