# Supplementary material for: Calycosin inhibits the in vitro and in vivo growth of breast cancer cells through WDR7-7-GPR30 Signaling
Source: J Exp Clin Cancer Res. 2017 Nov 2;36:153. doi: 10.1186/s13046-017-0625-y (PMC5667511; doi:10.1186/s13046-017-0625-y)
Supplement: Supplementary file 1 — The primer sequences. (DOCX 11 kb) [file 13046_2017_625_MOESM1_ESM.docx]

**Table S1. The primer sequences..**

GPR30: 5′-TCATTTCTGCCATGCACCCA-3′ and 5′-GTGGACAGGGTGTCTGATGT-3′

WDR7-7: 5′-AGCAGGGAAAGAATGGTCACA-3′ and

5′-TCAAACCCCTGTGGAATAAGTCT-3′

miR-375: 5′-CACAAAATTTGTTCGTTCGGCT-3′ and 5′-GTGCAGGGTCCGAGGT-3′

β-actin: 5′-TCACCAACTGGGACGACATG-3′ and 5′-GTCACCGGAGTCCATCACGAT-3′

U6: 5'-CTCGCTTCGGCAGCACA-3' and 5'-AACGCTTCACGAATTTGCGT-3'
