# Supplementary material for: Calycosin inhibits the in vitro and in vivo growth of breast cancer cells through WDR7-7-GPR30 Signaling
Source: J Exp Clin Cancer Res. 2017 Nov 2;36:153. doi: 10.1186/s13046-017-0625-y (PMC5667511; doi:10.1186/s13046-017-0625-y)
Supplement: Supplementary file 2 — The effects of calycosin treatment on lncRNA profiles in SKBR3 cells. (DOC 37 kb) [file 13046_2017_625_MOESM2_ESM.doc]

**Supplementary Table S2. The effects of calycosin treatment on lncRNA profiles in SKBR3 cells.**

| NO. | P.Value Gene_symbol | | Log 2 ratio |
| --- | --- | --- | --- |
|  |  |  | SKBR3 |
| 1 | 3.93939E-05 | WDR7-7 | 4.02 |
| 2 | 1.10878E-06 | TTC21B-AS1 | 3.93 |
| 3 | 0.001364491 | CTA-384D8.34 | 3.87 |
| 4 | 4.02429E-06 | RP11-686M17.5 | 3.64 |
| 5 | 1.88573E-05 | CTD-3046C4.1 | 3.51 |
| 6 | 0.000155782 | CTA-398F10.1 | 3.48 |
| 7 | 5.29702E-05 | RP11-686D22.9 | 3.38 |
| 8 | 2.7412E-05 | CTD-3131K8.2 | 2.73 |
| 9 | 0.000714287 | AC006262.5 | 2.52 |
| 10 | 1.55695E-05 | RP1-20C7.6 | 2.43 |
