# Supplementary figures and images for: Calycosin inhibits the in vitro and in vivo growth of breast cancer cells through WDR7-7-GPR30 Signaling
Source: J Exp Clin Cancer Res. 2017 Nov 2;36:153. doi: 10.1186/s13046-017-0625-y (PMC5667511; doi:10.1186/s13046-017-0625-y)

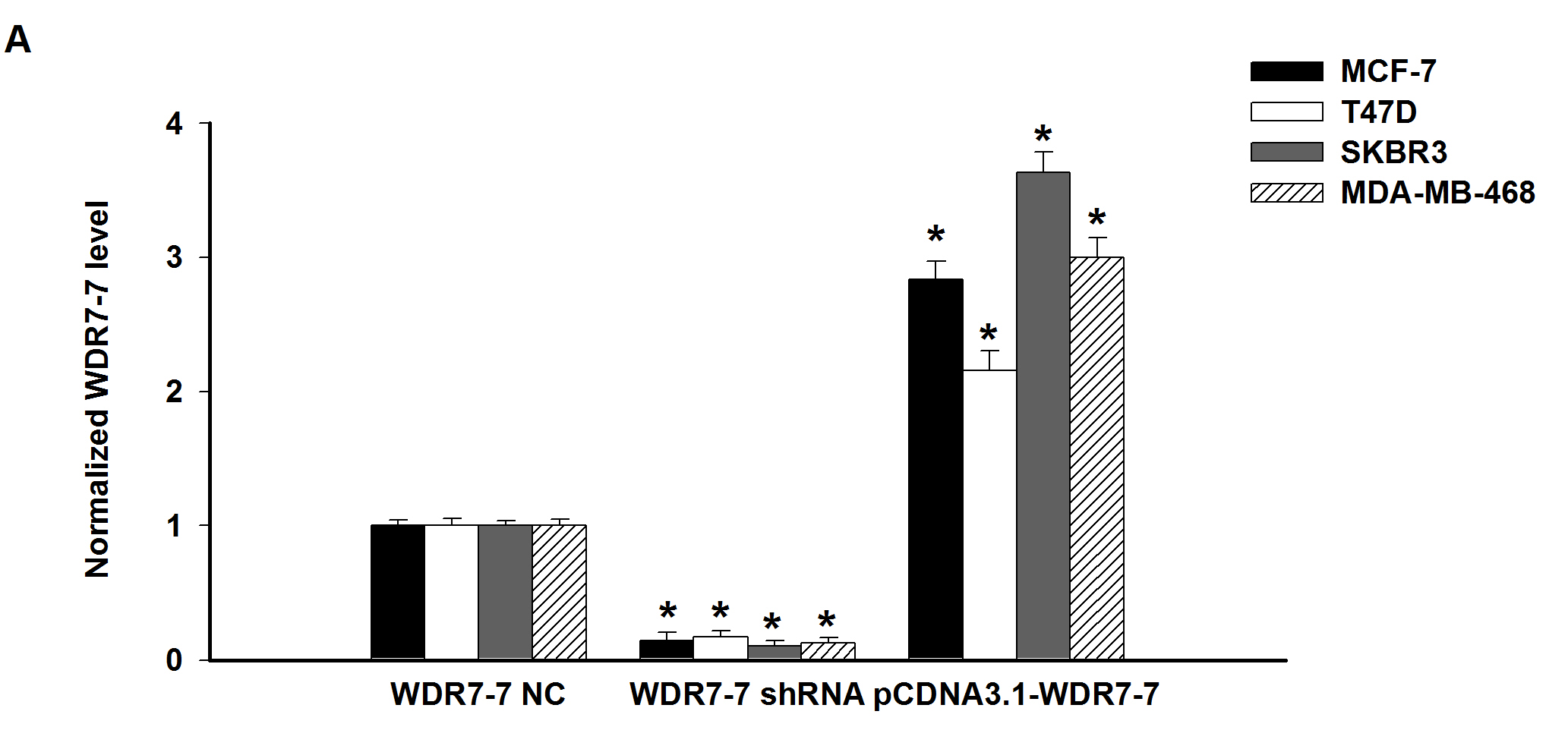

Supplement: Supplementary file 3 — Relative WDR7-7 expression in breast cancer cells transfected with WDR7-7 control, WDR7-7 shRNA, or pCDNA3.1-WDR7-7 vector. Representative data from three independent experiments are shown. *p < 0.05 vs. WDR7-7 control. (JPEG 297 kb) [file 13046_2017_625_MOESM3_ESM.jpg]

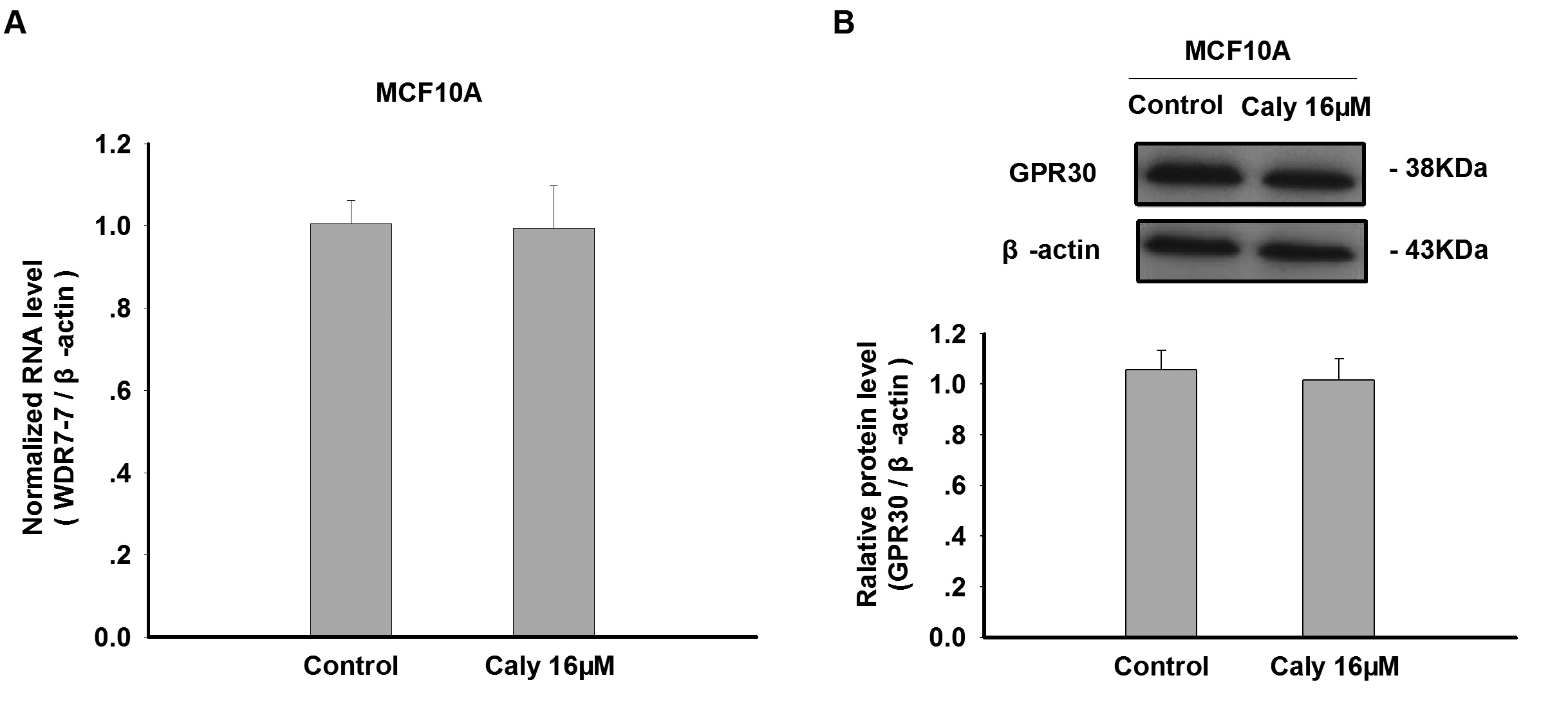

Supplement: Supplementary file 4 — The effects of calycosin on WDR7-7 and GPR30 expression in MCF10A cells. MCF10A cells were treated for 48 h with calycosin (0, 16 μM). (A) The transcript expression levels of WDR7-7 were determined using qRT-PCR with β-actin as the internal control. (B) The protein expression levels of GPR30 were determined using Western blotting and were normalized to those of β-actin. Representative data from three independent experiments are shown. (JPEG 267 kb) [file 13046_2017_625_MOESM4_ESM.jpg]

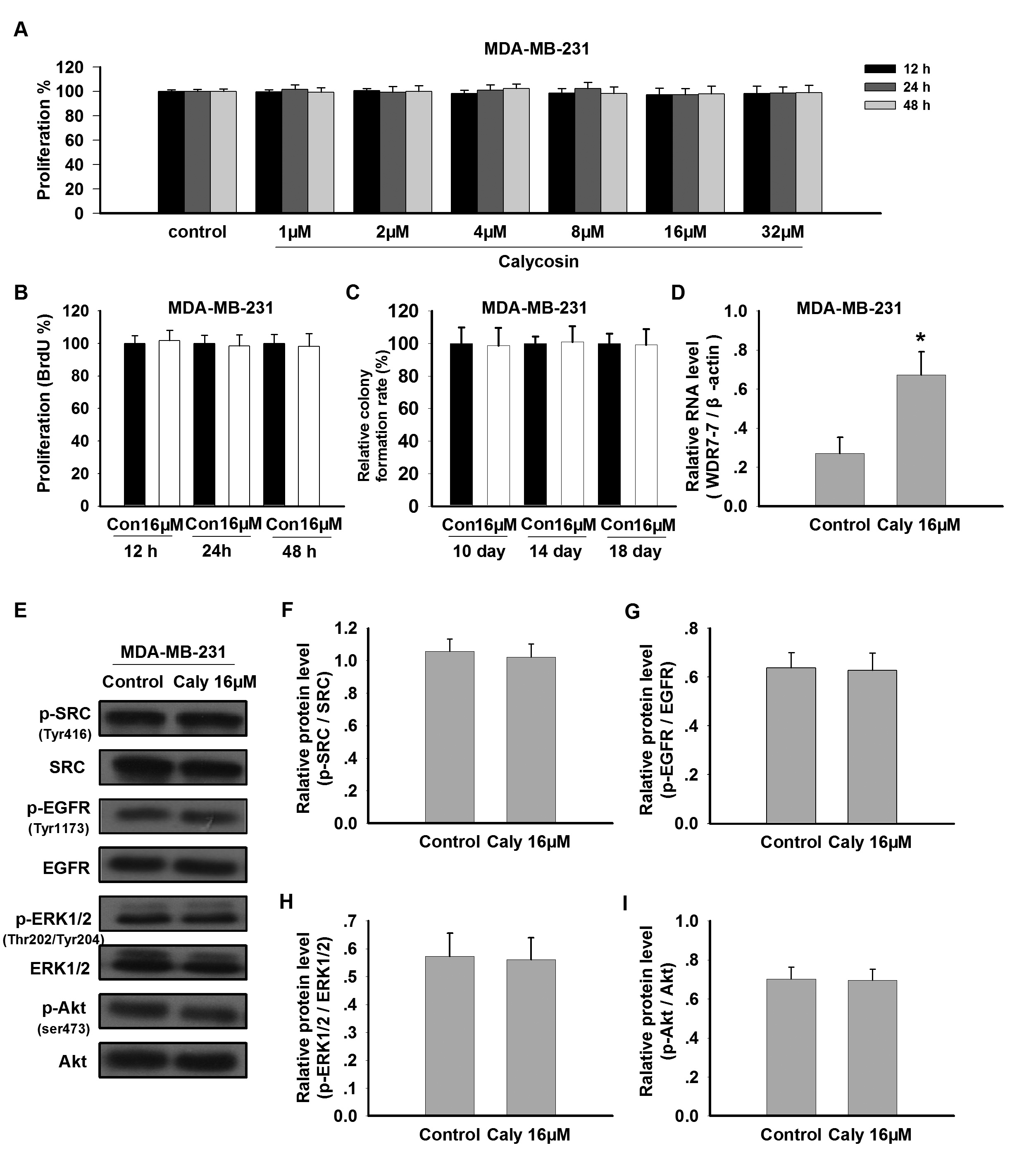

Supplement: Supplementary file 5 — The effect of calycosin on the proliferation of MDA-MB-231 breast cancer cells and WDR7-7-GPR30 signaling. MDA-MB-231 cells were treated for 12, 24, or 48 h with calycosin (1–32 μM); then, cell proliferation was quantified using (A) the CCK-8 assay, (B) BrdU assay, and (C) colony formation assay. (D) The transcript expression levels of WDR7-7 were determined using qRT-PCR with β-actin as the internal control. (E-I) The phosphorylation levels of SRC, EGFR, ERK1/2, and Akt were determined using Western blotting with the corresponding total protein as the internal control. The results are from three independent experiments that were each conducted in triplicate. *p < 0.05 vs. control (0 μM). (JPEG 892 kb) [file 13046_2017_625_MOESM5_ESM.jpg]
